# Contextual fear conditioning in virtual reality is affected by *5HTT*LPR and *NPSR1* polymorphisms: effects on fear-potentiated startle

**DOI:** 10.3389/fnbeh.2013.00031

**Published:** 2013-04-23

**Authors:** Evelyn Glotzbach-Schoon, Marta Andreatta, Andreas Reif, Heike Ewald, Christian Tröger, Christian Baumann, Jürgen Deckert, Andreas Mühlberger, Paul Pauli

**Affiliations:** ^1^Department of Psychology I, Biological Psychology, Clinical Psychology, and Psychotherapy, University of WürzburgWürzburg, Germany; ^2^Department of Psychiatry, Psychosomatics, and Psychotherapy, University of WürzburgWürzburg, Germany; ^3^Department of Psychology, Chair of Clinical Psychology, and Psychotherapy, University of RegensburgRegensburg, Germany

**Keywords:** *5HTT*LPR, *NPSR1*, gene × gene interaction, contextual fear conditioning, fear-potentiated startle

## Abstract

The serotonin (5-HT) and neuropeptide S (NPS) systems are discussed as important genetic modulators of fear and sustained anxiety contributing to the etiology of anxiety disorders. Sustained anxiety is a crucial characteristic of most anxiety disorders which likely develops through contextual fear conditioning. This study investigated if and how genetic alterations of the 5-HT and the NPS systems as well as their interaction modulate contextual fear conditioning; specifically, function polymorphic variants in the genes coding for the 5-HT transporter (*5HTT*) and the NPS receptor (*NPSR1*) were studied. A large group of healthy volunteers was therefore stratified for *5HTT*LPR (S+ vs. LL carriers) and *NPSR1* rs324981 (T+ vs. AA carriers) polymorphisms resulting in four genotype groups (S+/T+, S+/AA, LL/T+, LL/AA) of 20 participants each. All participants underwent contextual fear conditioning and extinction using a virtual reality (VR) paradigm. During acquisition, one virtual office room (anxiety context, CXT+) was paired with an unpredictable electric stimulus (unconditioned stimulus, US), whereas another virtual office room was not paired with any US (safety context, CXT−). During extinction no US was administered. Anxiety responses were quantified by fear-potentiated startle and ratings. Most importantly, we found a gene × gene interaction on fear-potentiated startle. Only carriers of both risk alleles (S+/T+) exhibited higher startle responses in CXT+ compared to CXT−. In contrast, anxiety ratings were only influenced by the *NPSR1* polymorphism with AA carriers showing higher anxiety ratings in CXT+ as compared to CXT−. Our results speak in favor of a two level account of fear conditioning with diverging effects on implicit vs. explicit fear responses. Enhanced contextual fear conditioning as reflected in potentiated startle responses may be an endophenotype for anxiety disorders.

## Introduction

Associative learning processes are crucial for the development of anxiety disorders (Mineka and Zinbarg, 2006). Cued fear conditioning which is a simple form of associative learning is regarded as a model for phasic fear and phobias (Grillon, 2002). In cued fear conditioning, a discrete cue (conditioned stimulus, CS) is predictably paired with an aversive event (unconditioned stimulus, US). By contrast, contextual fear conditioning may serve as a model for sustained and chronic anxiety because the US is not time-bound to a specific cue and is, therefore, experienced as an unpredictable event (Grillon, 2008). Animal and human studies demonstrate that sustained fear responses to contexts associated with unpredictable stressors are mediated by the amygdala, specifically by the bed nucleus of the stria terminalis (BNST) and the hippocampus (Alvarez et al., 2008, 2011; Marschner et al., 2008; Barot et al., 2009; Luyten et al., 2011). Importantly, it has been suggested that increased contextual anxiety elicited by unpredictable aversive events may be an important pathogenic marker for panic disorder and post-traumatic stress disorder (PTSD) (Grillon et al., 2008, 2009).

Although, threatening experiences and life stress have been reported to increase the risk for anxiety disorders (Watanabe et al., 2005; Melchior et al., 2007), the effect of environmental stress is also moderated by a genetic diathesis (Nugent et al., 2011). Supporting this view, a genetic contribution to the etiology of anxiety disorders (Gordon and Hen, 2004; Leonardo and Hen, 2006) and to fear conditioning (Merrill et al., 1999; Hettema et al., 2003; Lonsdorf and Kalisch, 2011) has been demonstrated. Especially, a polymorphism within the promoter region of the serotonin transporter (5-HTT) gene (*SLC6A4, 5HTT*) located on chromosome 17q11.1-q12 has been shown to play an important role in trait anxiety and anxiety disorders (Lesch et al., 1996; Amstadter et al., 2009; Skelton et al., 2012). The short (S) allele of the *5HTT* gene polymorphism (*5HTTLPR*) results in less expression of *5HTT* mRNA presumably leading to reduced serotonin reuptake compared to the long (L) variant (Hariri and Holmes, 2006). The S allele is associated with high trait anxiety and heightened amygdala activation toward emotional stimuli (Hariri et al., 2002; Heinz et al., 2005; Canli and Lesch, 2007; Dannlowski et al., 2010). Studies on cued fear conditioning using startle reflex as an indicator of learned fear reveal stronger fear conditioning in S compared to LL allele carriers (Lonsdorf et al., 2009; Klumpers et al., 2012), though the down regulation of fear after the offset of a fear cue (CS+) is not affected by the *5HTT*LPR polymorphism (Klumpers et al., 2012). These findings suggest that S allele carriers are characterized by faster fear learning and/or stronger fear reactivity than LL allele carriers, but fear regulation does not seem to be influenced by this genotype. Interestingly, the extinction of fear-potentiated startle in S allele carriers is additionally influenced by the *COMT*val158met polymorphism of the catechol-*O*-methyltransferase gene (*COMT*). Only those S allele carriers who additionally carried two met alleles (met/met) of the *COMT*val158met polymorphism exhibit enhanced startle responses to CS+ during extinction, which demonstrates a gene × gene interaction implicated in fear extinction (Lonsdorf et al., 2009).

The recently discovered neuropeptide S (NPS) and its receptor (NPSR) also seem to impact arousal, fear, and anxiety responses. *NPSR* mRNA has been found to be highly expressed in the amygdala, hippocampus and paraventricular hypothalamic nucleus in the rat brain (Xu et al., 2007; Jüngling et al., 2008). NPS binding to its receptor leads to increased glutamatergic transmission to intercalated GABAergic neurons in the amygdala (Jüngling et al., 2008). In rodents, NPS injection is found to have anxiolytic effects namely the reduction of contextual anxiety, cued fear, and enhancement of fear extinction (Jüngling et al., 2008; Meis et al., 2008; Fendt et al., 2010; Pape et al., 2010). A single nucleotide polymorphism (SNP; rs324981) in the human NPS receptor gene, *NPSR1*, leads to an amino-acid exchange from Asn to Ile at position 107 of the protein resulting in potentiated efficacy of NPS at NPSR in the T allele (Ile107) compared to the A allele (Asn107) carriers (Reinscheid et al., 2005). Studies in humans suggest that rs324981 is associated with anxiety disorders, as the more active T allele is associated with panic disorder in females (Domschke et al., 2011). In healthy volunteers, T allele carriers exhibit increased basolateral amygdala activation to fearful faces (Dannlowski et al., 2011), and report generally enhanced fear ratings to both a fear (CS+) and a safety signal (CS−) during a cued fear conditioning paradigm thus T and homozygous AA carriers do not differ in differential fear learning (Raczka et al., 2010).

In conclusion, both the S and the T alleles of the *5HTT*LPR and *NPSR1* polymorphisms, seem to enhance the vulnerability to anxiety levels and/or anxiety disorders, but only the S allele influences differential cued fear conditioning (Lonsdorf et al., 2009; Raczka et al., 2010). However, while cue conditioning is a good model for phobic fear, contextual fear learning is a better model for sustained anxiety, and recent animal studies suggest the importance of these two polymorphisms for contextual fear conditioning. For instance, *5HTT* knockout mice display enhanced contextual fear conditioning and impaired fear extinction compared to wild-type mice (Dai et al., 2008), and *NPSR1* knockout mice exhibit enhanced freezing to a fear context (Fendt et al., 2011).

The present study is designed to examine a gene × gene interaction of *5HTT*LPR and *NPSR1* polymorphisms on contextual fear conditioning and extinction. We use a virtual reality (VR) paradigm with two virtual office rooms serving as conditioned contexts (Glotzbach et al., 2012; Tröger et al., 2012). Importantly, we assess fear responses on a verbal (ratings), a behavioral (fear-potentiated startle), and a physiological level (skin conductance). A valid behavioral measure of fear and anxiety which can be used across species is the fear-potentiated startle response (Fendt and Fanselow, 1999; Blumenthal et al., 2005). Startle responses, which can be measured in humans by means of an electromyogram of the M. *orbicularis oculi* (Blumenthal et al., 2005), are potentiated by influences of the central amygdala (CeA) on the caudal pontine reticular nucleus (PnC) (for reviews see Koch, 1999; Davis, 2006). Thus, negative, threatening, and fear inducing events lead to startle potentiation (Lang et al., 1990). As the fear-potentiation of the startle reflex occurs without cortical processes, it is thought to be an implicit measure of fear which is greatly independent of cognitive processes (Hamm et al., 2003; Hamm and Weike, 2005). In contrast, fear ratings are considered an explicit measure of fear, and skin conductance is considered a physiological measure of arousal (Bradley and Lang, 2007).

To disentangle genetic contributions to contextual fear conditioning, we here specifically probe a potential gene × gene interaction of *5HTT*LPR and *NPSR1* and hypothesize that carriers of both risk alleles (S and T) are characterized by an enhanced acquisition of contextual anxiety compared to no-risk allele carriers (LL or AA).

## Materials and methods

### Sample

Ninety-three (Caucasian descent, 60 female; mean age 23.96 years, *SD* = 3.14) healthy subjects were drawn from a larger sample (*N* = 497) ascertained within the framework of the collaborative research center SFB TRR 58 (Domschke et al., 2012). For genotyping, a blood sample (18 ml EDTA blood) was collected from each participant. Participants were excluded if they had current or prior diagnosis of DSM-IV axis-I (using the Mini-International Neuropsychiatric Interview (MINI), Sheehan et al., 1998; German version: Ackenheil et al., 1999), any neurological or somatic disorder, illegal drug consumption (assessed by a urine drug screening for amphetamine, barbiturates, benzodiazepines, cocaine, ecstasy, methamphetamine, methadone, opiates, tricyclic antidepressants, tetrahydrocannabinol), alcohol consumption of more than 140 g per week, daily smoking of more than 20 cigarettes per day, daily use of any medication (except for hormonal contraception), pregnancy and left handedness. For the present study, we additionally excluded psychology students because of their familiarity with conditioning protocols.

Prior to genotyping, participants completed the Trait version of the State-Trait-Anxiety-Inventory (STAI; Spielberger et al., 1970; German version: Laux et al., 1981), the Anxiety-Sensitivity-Index (ASI; Reiss et al., 1986; German version: Alpers and Pauli, 2001), and the Behavioral Inhibition System and Behavioral Approach System (BIS-BAS; Carver and White, 1994; German version: Strobel et al., 2001). Life stress history was assessed with a 27-item self-report questionnaire regarding work, relocation and house renovation, financial and legal problems, own serious illness or of a friend or family member, physical or sexual abuse, etc. (see Canli et al., 2006; Herrmann et al., 2009). Participants had to indicate how many of these stressful life events they had experienced, and a sum score was calculated.

All participants gave their written informed consent. Participants gained €50 for their participation. The study was approved by the Ethics Committee of the Medical Faculty of the University of Würzburg. Thirteen participants had to be excluded because of technical problems (*n* = 7), low startle reactivity (*n* = 3), and excessive artifacts in startle data (*n* = 2; for startle response quantification see Data Reduction), and VR simulator sickness (*n* = 1). Thus, the final sample consisted of 80 participants.

### Genotyping

Subjects were genotyped for *5HTT*LPR and *NPSR* rs324981 A/T (Asn107Ile) polymorphisms as reported by Domschke et al. (2011) and Klauke et al. (2011). Subjects with one or two S alleles of the *5HTT*LPR polymorphism were grouped together (S+). Similarly, subjects with one or two T alleles of the *NPSR1* polymorphism (T+) were grouped like in previous studies (Hariri et al., 2002; Lonsdorf et al., 2009; Raczka et al., 2010; Domschke et al., 2011) resulting in the following four combined genotype groups: S+/T+, S+/AA, LL/T+, and LL/AA. The experimenter was blind to genotype.

### Stimuli, apparatus, and design

**Figure 1:**
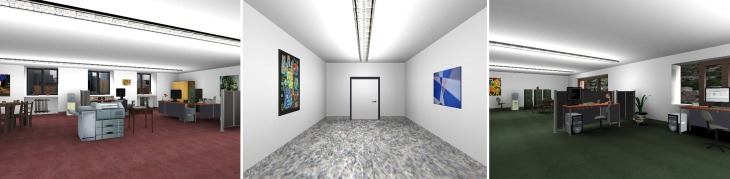
**Screenshots of the two office rooms and the connecting corridor (intertrial–interval, ITI).** During acquisition one office room was paired with mildly painful electrical stimuli (anxiety context, CXT+), whereas the other office room was never paired with electrical stimuli (safety context, CXT−).

A detailed description of the VR equipment, context stimuli, US, recording of physiological data, and procedure and design is published elsewhere (Tröger et al., 2012; Glotzbach-Schoon et al., 2013). In brief, the VR environment was created with the Source Engine (Valve Corporation, Bellevue, USA). Two different virtual office rooms served as different contexts (Figure 1). The VR environment, instructions, and ratings were presented with a Z800 3D Visor head-mounted display (HMD; eMagin, Hopewell Junction, USA). The head position was monitored by an electromagnetic tracking device (Patriot, Polhemus Corp., Colchester, USA) in order to adapt the field of view to head movements and to assess head orientation. The experimental procedure was controlled by the software Cyber Session (version 5.3.38), developed in the Department of Psychology I, University of Würzburg.

The US was an electric stimulus generated by a constant current stimulator (Digitimer DS7A, Digitimer Ltd., Welwyn Garden City, UK) and triggered with a frequency of 50 Hz and a duration of 200 ms by the software Cyber Session. The electric stimulus was applied by a surface electrode placed on the dominant forearm. The intensity of the current was individually adjusted to each participant's pain threshold as done previously (Andreatta et al., 2010) and increased by 30% to avoid habituation. Neither current intensity nor pain ratings of the US (on a scale with anchors at 0 = *no feeling at all*, 4 = *just noticeable pain*, and 10 = *very strong pain*) were influenced by genotype (all *p*s > 0.2; see Table 1). However, there was a group effect of *NPSR1* polymorphism on US arousal (on a scale from 0 = *no arousal at all* to 100 = *very high arousal*) and a trend for US valence rating (on a scale from 0 = *very negative* to 100 = *very positive*). AA carriers rated the US as more arousing (*M* = 54.40, *SD* = 21.99), *F*_(1, 76)_ = 5.34, *p* = 0.024, η^2^_*p*_ = 0.07, and by trend as more negative (*M* = 35.25, *SD* = 16.52), *F*_(1, 76)_ = 3.40, *p* = 0.069, η^2^_*p*_ = 0.04, than T+ carriers (arousal: *M* = 42.62, *SD* = 23.37; valence: *M* = 41.88, *SD* = 15.43).

**Table 1:** **Demographic and psychometric data for genotype groups**.

***NPSR1***	***5HTT*LPR**		**Total**
	**S+**	***LL***	
T+	10 female, 10 male	11 female, 9 male	21 female, 19 male
	Age = 24.05 years (2.46)	Age = 24.20 years (4.43)	Age = 24.13 years (3.54)
	STAI Trait = 34.20 (6.61)	STAI Trait = 32.50 (7.33)	**STAI Trait = 33.35 (6.95)**
	ASI = 15.05 (6.25)	ASI = 13.65 (7.71)	ASI = 14.35 (6.97)
	BIS = 19.25 (2.59)	BIS = 18.20 (4.65)	BIS = 18.73 (3.76)
	BAS = 43.60 (3.95)	BAS = 41.85 (5.42)	BAS = 42.73 (4.77)
	Current intensity = 2.85 mA (1.26)	Current intensity = 2.96 mA (1.99)	Current intensity = 2.91 mA (1.64)
	US pain rating = 5.10 (1.07)	US pain rating = 5.00 (0.92)	US pain rating = 5.05 (0.99)
	*n* = 20	*n* = 20	*n* = 40
AA	13 female, 7 male	15 female, 5 male	28 female, 12 male
	Age = 23.50 years (2.65)	Age = 24.35 years (3.75)	Age = 23.92 years (3.23)
	STAI Trait = 36.70 (6.73)	STAI Trait = 36.35 (7.34)	**STAI Trait = 36.53 (6.95)**
	ASI = 15.30 (7.12)	ASI = 16.75 (7.68)	ASI = 16.03 (7.34)
	BIS = 19.30 (3.05)	BIS = 20.05 (2.98)	BIS = 19.68 (3.00)
	BAS = 41.90 (3.89)	BAS = 42.60 (3.22)	BAS = 42.25 (3.54)
	Current intensity = 2.72 mA (1.30)	Current intensity = 2.28 mA (1.04)	Current intensity = 2.50 mA (1.18)
	US pain rating = 4.90 (0.85)	US pain rating = 5.20 (1.61)	US pain rating = 5.05 (1.28)
	*n* = 20	*n* = 20	*n* = 40
Total	23 female, 17 male	26 female, 14 male	49 female, 31 male
	Age = 23.78 years (2.54)	Age = 24.28 years (4.05)	Age = 24.03 years (3.37)
	STAI Trait = 36.70 (6.73)	STAI Trait = 36.35 (7.34)	STAI Trait = 34.94 (7.09)
	ASI = 15.18 (6.61)	ASI = 15.20 (7.76)	ASI = 15.19 (7.16)
	BIS = 19.28 (2.79)	BIS = 19.13 (3.97)	BIS = 19.20 (3.41)
	BAS = 42.75 (3.97)	BAS = 42.23 (4.42)	BAS = 42.49 (4.18)
	Current intensity = 2.72 mA (1.30)	Current intensity = 2.28 mA (1.04)	Current intensity = 2.50 mA (1.18)
	US pain rating = 5.00 (0.96)	US pain rating = 5.10 (1.30)	US pain rating = 5.05 (1.14)
	*n* = 40	*n* = 40	*n* = 80

Frequencies and Means (SD). Significant group differences are displayed in bold. STAI, State-Trait-Anxiety-Inventory; ASI, Anxiety Sensitivity Index; BIS, Behavior Inhibition Scale; BAS, Behavior Avoidance Scale; US, Unconditioned Stimulus.

Startle probes of 50 ms, 103 dB (A) white noise were presented for physiological measures. Startle reflex was measured by electromyographic activity (EMG) from the M. *orbicularis oculi* with electrodes placed centrally under and next to the lateral canthus of the left eye. Ground and reference electrodes were placed at the mastoids. Impedances were kept below 10 kΩ. The EMG signal was filtered online with a 50 Hz notch filter and sampled at 1000 Hz. At the beginning of the experiment, four startle tones were presented at intervals of 15–17 s to reduce the initial startle reactivity. Skin conductance level (SCL) was measured on the thenar of the nondominant hand by two Ag-AgCl electrodes. Physiological data were recorded by Vision Recorder software (Brain Products Inc., Munich, Germany).

**Figure 2:**
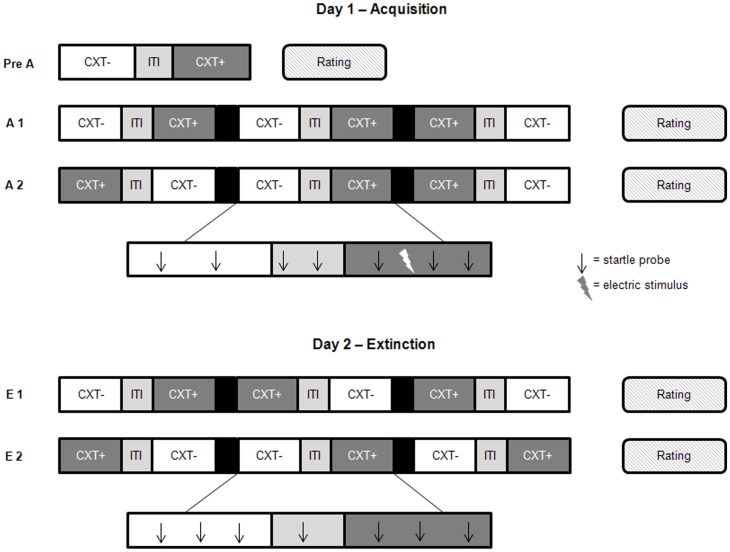
**Design of the contextual fear conditioning paradigm.** Day 1 started with a pre-acquisition phase (Pre A) in which participants actively explored each context once by using a joystick. During the acquisition phases, one to three electric stimuli (US) per visit were presented in the anxiety context (CXT+). In the safety context (CXT−) no electric stimuli were administered. During extinction on Day 2, no electric stimuli were given. The acquisition phases as well as the extinction phases consisted of two blocks each (A1 and A2, E1 and E2). Participants were passively guided through the contexts on pre-recorded paths. They always enter one context, were then guided through the corridor (intertrial-interval, ITI) into the other context. After the consecutive presentation of the three conditions, the display turned black. This procedure was repeated three times during one block. Two to three startle probes were presented during each context presentation, and one to two startle probes during the ITI. After each phase, ratings were obtained. The order of the presentation of the different contexts was counterbalanced across participants and genotype groups.

The experiment was run on two consecutive days separated by 24 h (see Figure 2). Two acquisition phases (Acquisition 1, Acquisition 2) were performed on Day 1 with US administered only in one office room (anxiety context, CXT+) but not in the other (safety context, CXT−). The corridor served as a control context and as an intertrial–interval (ITI) between CXT+ and CXT− during each run. On Day 2, two extinction phases (Extinction 1, Extinction 2) were conducted, where no US was presented. Before the experimental sessions of each day, participants were required to complete the State version of the STAI and the Positive And Negative Affect Schedule (PANAS; Watson et al., 1988; German version: Krohne et al., 1996). Day 1 started with a pre-acquisition phase; participants explored each virtual office room for 2 min by actively moving themselves through the VR using a joystick. Subsequently, two successive acquisition phases were started with each phase consisting of three presentations per context category (CXT+, CXT−, ITI). Participants were passively moved through the VR environment i.e., they could not influence the way through the office rooms and corridor. However, participants were always able to adapt their line of sight in the VR by head movements. The passages through CXT+ and CXT− lasted about 85 s each; the ITI passage lasted about 35 s. Fifteen startle probes were presented per context and nine startle probes during ITI at intervals of 10–34 s. Participants received 1–3 mildly painful electric stimuli in CXT+ but never in CXT−, resulting in a total of 12 electric stimuli during acquisition. The US was unpredictably presented at different locations in CXT+ preventing participants from associating specific cues within this context with shock administration. The office rooms were randomly assigned to the two conditions (CXT+ vs. CXT−) and counterbalanced across participants and groups. The sequence of context presentations was pseudo-random and also counterbalanced across participants and groups. Before the first acquisition phase, participants were instructed to figure out the relationship between the context and the US (Schiller et al., 2010). The experimental session on Day 2 was nearly the same. All electrodes were attached, including the one for the US presentation. Two extinction blocks were conducted where no US was administered. Like on Day 1, each block consisted of three runs where participants were passively moved through each context once. The same number of startle tones was presented during CXT+, CXT−, and ITI presentations as on Day 1.

Ratings for anxiety and US-expectancy for the two conditioned contexts (CXT+, CXT−) were obtained after the different phases of the experiment regarding the previously experienced phase (Day 1: pre-acquisition, Acquisition 1, Acquisition 2; Day 2: Extinction 1, Extinction 2). Rating scales ranged from 0 (*no anxiety at all*/*no expectancy at all*) to 100 (*very high anxiety*/*definitely expected*). Awareness of the CXT+ US contingency was assessed with an open question (“In which room did you receive electrical stimuli?”) after Acquisition 1 and 2 of Day 1 and participants had to describe the room. If participants described only the CXT+ they were labeled as “aware,” whereas if they stated that in both contexts any US was administered (CXT+ and CXT−) they were labeled as “uncertain.” In total, there were nine uncertain participants who were equally distributed over *5HTT*LPR (S+: *n* = 5; LL: *n* = 4), χ^2^(1, *N* = 80) = 0.13, *p* = 0.723 and *NPSR1* genotype groups (T+: *n* = 5; AA: *n* = 4), χ^2^(1, *N* = 80) = 0.13, *p* = 0.723.

### Data reduction

#### Startle response

Eyeblink EMG Data were processed with Vision Analyzer software (Brain Products Inc., Munich, Germany). The signal of orbital electrodes was filtered offline with a 500 Hz High Cut off and a 30 Hz Low Cut off Filter. The signal was rectified, smoothed (50 ms moving window average), and baseline corrected (50 ms before startle probe onset). The peak magnitude was identified within a time window from 20 to 200 ms after the probe onset. Artifact rejection was made by hand through excluding responses with baseline shifts above or below 5 μV and pre-blinks 50 ms before probe onsets higher than 5 μV. Magnitudes smaller than 5 μV were coded as zero. Responders vs. non-responders were defined on the basis of sufficient valid responses, which were artifact free and higher than 5 μV. If there were less than two valid responses per stimulus category (CXT+, CXT−, ITI) in a given phase (Acquisition 1, Acquisition 2, Extinction 1, and Extinction 2), the participant was excluded from further analysis. There were 5 participants who were excluded due to these criteria. Magnitudes in the acquisition and extinction phases were standardized into *T*-scores for each participant.

#### Skin conductance level

SCL data was filtered with 1 Hz High Cut-off. The mean tonic SCL was computed over each context presentation (excluding epochs from US presentation to 10 s after US presentation to avoid an increased SCL due to US presentation). SCL data were log-transformed [log_10_(SCL+1)] to normalize the distribution.

#### Statistical analysis

Prior to statistical analysis physiological data were averaged for each phase (Acquisition 1, Acquisition 2, Extinction 1, and Extinction 2) across three runs. Fear-potentiated startle was determined as the difference score between the mean startle response during contexts and ITI (CXT+ -ITI, or CXT− -ITI). During pre-acquisition, SCL data were assessed with a 2 (Context: CXT+, CXT−) × 2 (*5HTT*LPR: S+, LL) × 2 (*NPSR1*: T+, AA) Analysis of Variance (ANOVA). Acquisition and extinction data were analyzed separately with 2 (Context: CXT+, CXT−) × 2 (Phase: 1, 2) × 2 (*NPSR1*: T+, AA) × 2 (*5HTT*LPR: S+, LL) ANOVAs, respectively. To clarify significant main effects or interactions, *F* contrasts were calculated. In all analyzes, the alpha level was set at *p* ≤ 0.05. Effect sizes were calculated using the partial eta (η^2^_*p*_). On Day 1, rating data after pre-acquisition and acquisition phases of one participant (LL/T+) were missing due to technical problems.

## Results

### Sample characteristics

The final sample consisted of 80 participants with 20 participants per combined genotype group (S+/T+, S+/AA, LL/T+, LL/AA). There were less homozygous, SS (*n* = 14) or TT (*n* = 15), carriers than heterozygous, SL (*n* = 26) or TA (*n* = 25), carriers. However, homozygous SS carriers were equally distributed over *NPSR1* subgroups (SS/AA: *n* = 6; SS/TA: *n* = 5; SS/TT: *n* = 3), and homozygous TT carriers were equally distributed over *5HTT*LPR subgroups (SS/TT: *n* = 3; SL/TT: *n* = 4; LL/TT: *n* = 8), χ^2^(4, *N* = 80) = 0.58, *p* = 0.97. Demographic and psychometric characteristics of genotype groups are displayed in Table 1. There were less male than female participants in the final sample (31 male, 49 female), but male participants were not statistically overrepresented in any *NPSR1*, χ^2^(1, *N* = 80) = 2.58, *p* = 0.108, or *5HTT*LPR genotype group, χ^2^(1, *N* = 80) = 0.47, *p* = 0.491, (see Table 1). Additionally, genotype groups did not differ in age, ASI, BIS, or BAS scores (all *p*s > 0.2). However, AA allele carriers of the *NPSR1* polymorphism reported higher trait anxiety than T+ allele carriers, *F*_(1, 76)_ = 4.10, *p* = 0.046, η^2^_*p*_ = 0.05.

State anxiety, negative affect, and positive affect were measured before each experimental session and analyzed with 2 (Day: 1, 2) × 2 (*5HTT*LPR: S+, LL) × 2 (*NPSR1*: T+, AA) ANOVAs. State anxiety and negative affect were not influenced by any genotype (all *p*s > 0.2). For positive affect, there was only a significant main effect of day, *F*_(1, 76)_ = 5.31, *p* = 0.024, η^2^_*p*_= 0.07, with higher positive affect on Day 1 (*M* = 29.89, *SD* = 6.11) compared to Day 2 (*M* = 28.76, *SD* = 6.93).

### Pre-acquisition

There were neither significant differences between contexts nor any effects of genotype during pre-acquisition in SCL data (all *p*s > 0.2) or in anxiety ratings (all *p*s > 0.5).

### Acquisition (Day 1)

#### Fear-potentiated startle

**Figure 3:**
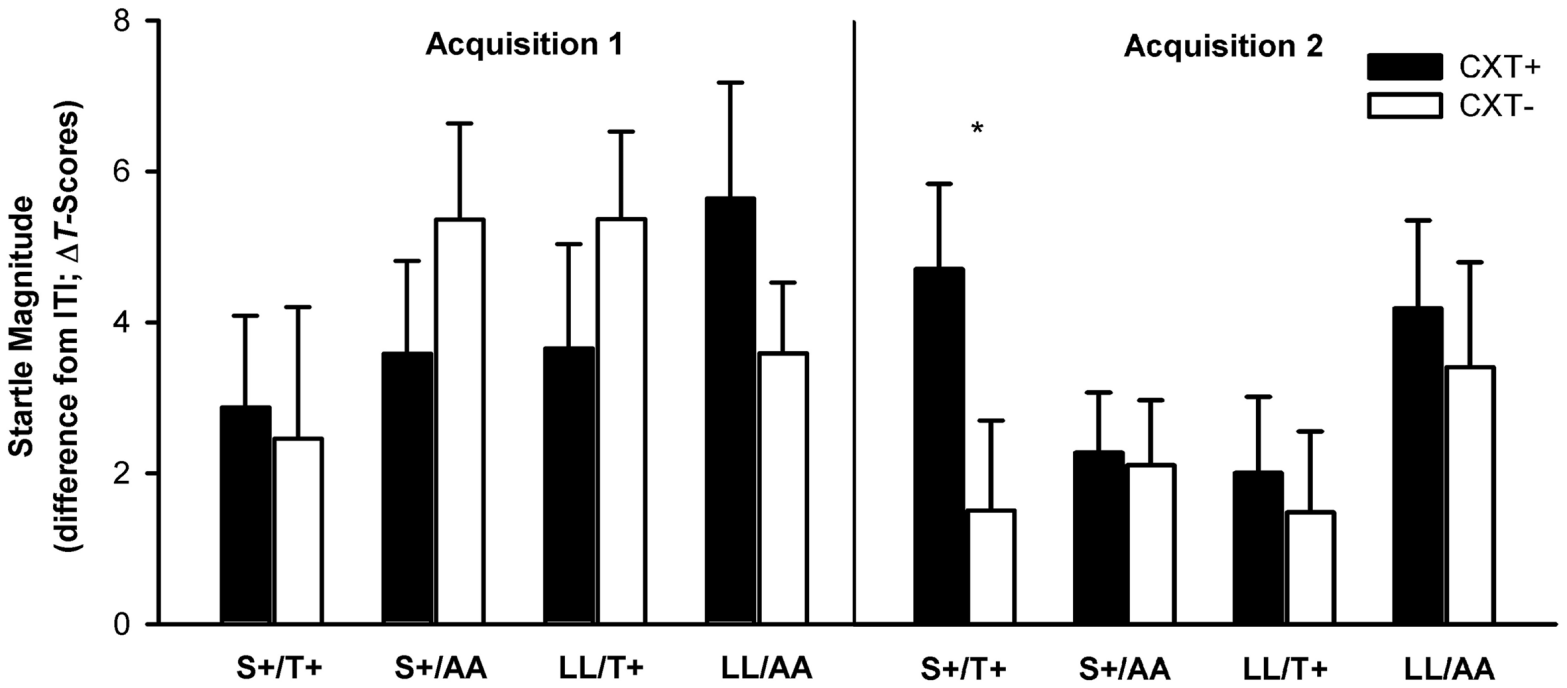
**Startle magnitudes during Acquisition 1 (left) and Acquisition 2 (right) on Day 1.** Black bars depict differences between startle magnitudes during CXT+ (anxiety context) and ITI (intertrial–interval, corridor). White bars depict differences between startle magnitudes during CXT− (safety context) and ITI. Results are shown separately for each combined genotype group of *5HTT*LPR (S+ vs. LL) and NPSR1 (T+ vs. AA) polymorphisms. Error bars represent standard error of the mean (SEM). ^*^p < 0.05.

Most important, the ANOVA revealed a significant three-way interaction of Context × *5HTT*LPR × *NPSR1*, *F*_(1, 76)_ = 7.00, *p* = 0.010, η^2^_*p*_ = 0.08. This interaction was driven by the fact that fear-potentiated startle in CXT+ compared to CXT− was only apparent in the carriers of both risk alleles, S+/T+, *F*_(1, 19)_ = 3.94, *p* = 0.062, η^2^_*p*_ = 0.17, whereas carriers of one risk allele (S+/AA, LL/T+) or of no risk allele (LL/AA) did not show differential contextual fear conditioning (all *p*s > 0.2). The marginal conditioning effect within the S+/T+ group was due to averaging startle responses across both acquisition phases. As characteristic for learning, the conditioning effect was significant during Acquisition 2, *F*_(1, 19)_ = 6.94, *p* = 0.016, η^2^_*p*_ = 0.27, but not during Acquisition 1, *F*_(1, 19)_ < 1 (see Figure 3). Moreover, another relevant outcome was a main effect of phase, *F*_(1, 76)_ = 3.74, *p* = 0.057, η^2^_*p*_ = 0.05, which just failed to reach the significance level indicating a habituation of startle responses from Acquisition 1 (*M* = 4.02, *SD* = 4.95) to Acquisition 2 (*M* = 2.69, *SD* = 4.24).

#### Skin conductance

**Figure 4:**
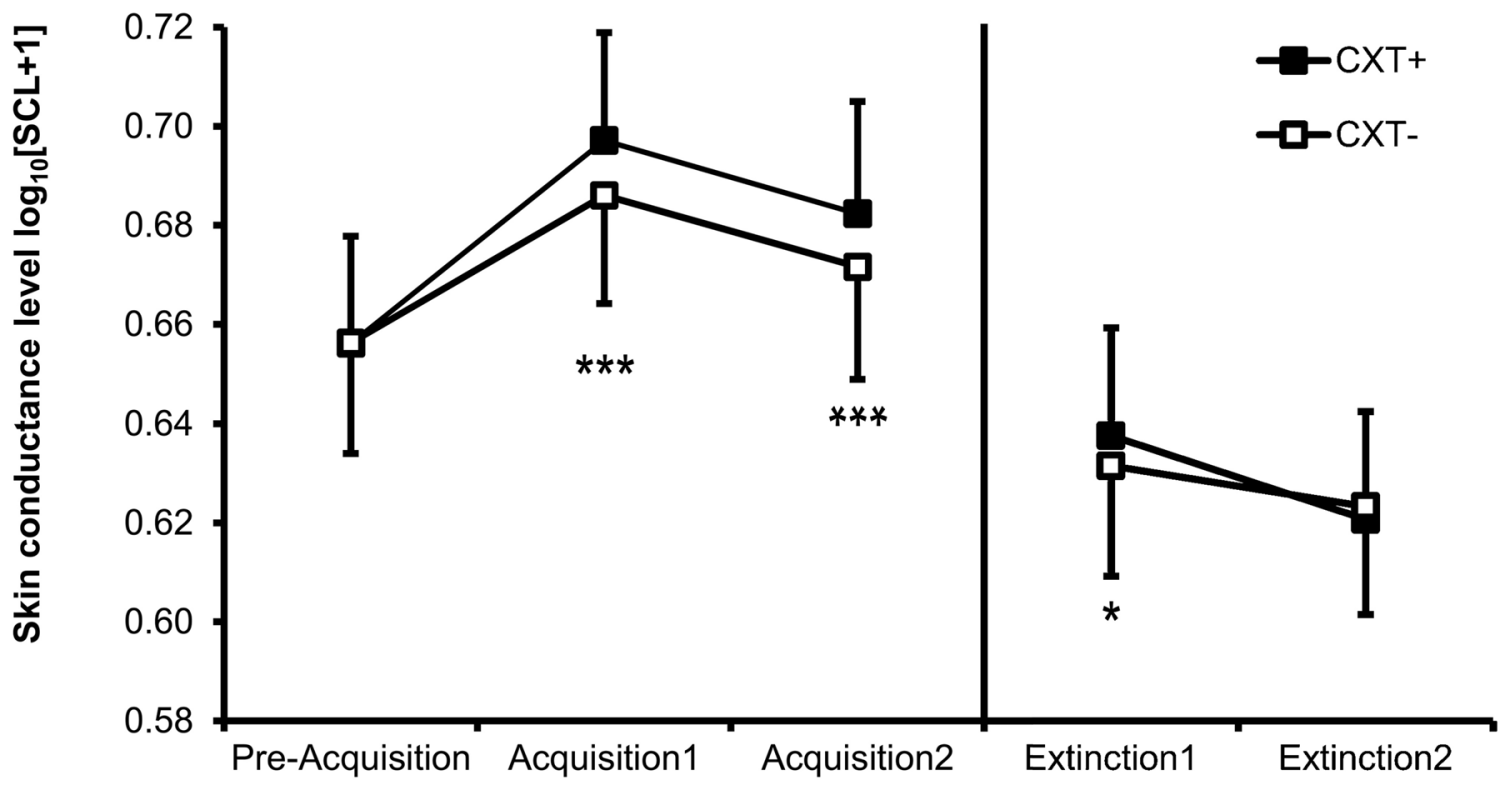
**Skin conductance level (SCL, log-transformed) of all participants during Day 1 (Pre-Acquisition, Acquisition 1 and 2) and Day 2 (Extinction 1 and 2).** Black bars depict SCL during CXT+ (anxiety context). White bars depict SCL during CXT− (safety context). Error bars represent standard error of the mean (SEM). ^*^p < 0.05, ^***^p < 0.001.

Successful contextual fear conditioning is reflected in a significant main effect of context, *F*_(1, 76)_ = 48.24, *p* < 0.001, η^2^_*p*_ = 0.39, with enhanced SCL in CXT+ (*M* = 0.690, *SD* = 0.198) compared to CXT− (*M* = 0.679, *SD* = 0.198) (see Figure 4). In addition, SCL habituated from Acquisition 1 (*M* = 0.691, *SD* = 0.195) to Acquisition 2 (*M* = 0.677, *SD* = 0.203), main effect of phase *F*_(1, 76)_ = 10.32, *p* = 0.002, η^2^_*p*_ = 0.12. None of the main or interaction effects involving a genotype reached significance (all *p*s > 0.1).

#### Anxiety rating

**Figure 5:**
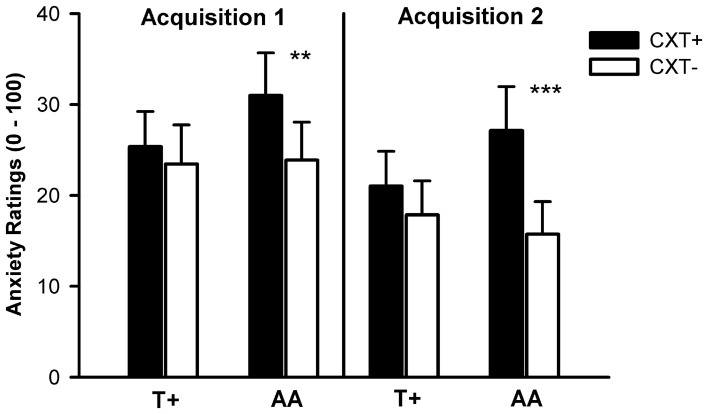
**Anxiety ratings from 0 (no anxiety at all) to 100 (very high anxiety) after Acquisition 1 (left) and Acquisition 2 (right) on Day 1.** Black bars depict ratings for CXT+ (anxiety context). White bars depict ratings for CXT− (safety context). Results are shown separately for *NPSR1* genotype groups (T+ vs. AA carriers). Error bars represent standard errors of the mean (SEM). ^**^p < 0.01, ^***^p < 0.001.

The ANOVA revealed significant main effects of context, *F*_(1, 75)_ = 14.21, *p* < 0.001, η^2^_*p*_ = 0.16, and phase, *F*_(1, 75)_ = 14.74, *p* < 0.001, η^2^_*p*_ = 0.16, as well as significant interactions of Context × *NPSR1*, *F*_(1, 75)_ = 5.67, *p* = 0.020, η^2^_*p*_ = 0.07, and Phase × *5HTT*LPR, *F*_(1, 75)_ = 7.05, *p* = 0.010, η^2^_*p*_ = 0.09. The main effect of context indicated successful contextual fear conditioning; the CXT+ was rated as more anxiety eliciting (*M* = 25.92, *SD* = 26.27) than the CTX- (*M* = 20.23, *SD* = 23.37) in all participants. The main effect of phase reflected an overall decrease of anxiety from Acquisition 1 (*M* = 25.70, *SD* = 25.69) to Acquisition 2 (*M* = 20.45, *SD* = 23.68). The Context × *NPSR1* interaction was due to the fact that AA carriers displayed learning. AA carriers reported higher anxiety in CXT+ compared to CXT−, *F*_(1, 39)_ = 15.65, *p* < 0.001, η^2^_*p*_ = 0.29, whereas T+ carriers did not, *F*_(1, 38)_ = 1.19, *p* = 0.281, η^2^_*p*_ = 0.03, as depicted in Figure 5. The conditioning effect in the AA group was not due to the fact that only the subgroup of S+/AA carriers exhibited higher anxiety ratings for CXT+ vs. CXT−, and not LL/AA carriers. Indeed, *5HTT*LPR polymorphism had no impact on conditioning of anxiety ratings. While both S+/AA (*p* = 0.003) and LL/AA (*p* = 0.039) carriers showed differential conditioning effects, both T+ allele groups i.e., S+/T+ (*p* = 0.961) and LL/T+ (*p* = 0.160), did not. *Post-hoc* tests regarding the Phase × *5HTT*LPR interaction revealed that anxiety ratings declined in LL carriers from Acquisition 1 (*M* = 30.71, *SD* = 26.20) to Acquisition 2 (*M* = 21.74, *SD* = 23.50), *F*_(1, 38)_ = 12.43, *p* = 0.001, η^2^_*p*_ = 0.25, but not in S+ carriers, *F*_(1, 39)_ = 2.41, *p* = 0.128, η^2^_*p*_ = 0.06, (Acquisition 1: *M* = 20.81, *SD* = 24.52; Acquisition 2: *M* = 19.19, *SD* = 24.10).

#### US-expectancy rating

**Figure 6:**
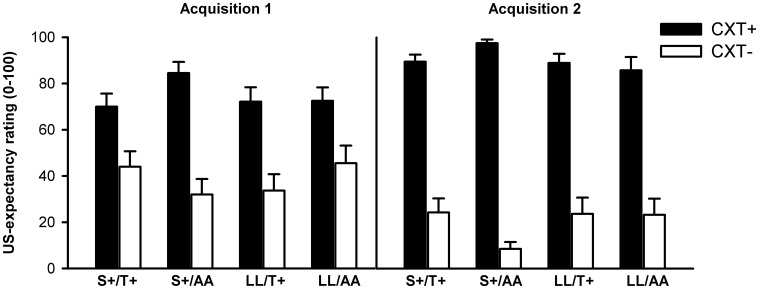
**US-expectancy ratings from 0 (not expected at all) to 100 (definitely expected) after Acquisition 1 (left) and Acquisition 2 (right) on Day 1.** Black bars depict ratings for CXT+ (anxiety context). White bars depict ratings for CXT− (safety context). Results are shown separately for each combined genotype group of *5HTT*LPR (S+ vs. LL) and *NPSR1* (T+ vs. AA) polymorphisms. Error bars represent standard error of the mean (SEM).

There was a significant main effect of context, *F*_(1, 75)_ = 246.48, *p* < 0.001, η^2^_*p*_ = 0.77, and significant interactions of Phase × Context, *F*_(1, 75)_ = 56.64, *p* < 0.001, η^2^_*p*_ = 0.43, and Context × *NPSR1* × *5HTT*LPR, *F*_(1, 75)_ = 5.64, *p* = 0.020, η^2^_*p*_ = 0.07. After Acquisition 1 and 2, all participants rated the expectancy of receiving a US in the CXT+ (Acquisition 1: *M* = 74.81, *SD* = 25.22; Acquisition 2: *M* = 90.44, *SD* = 17.45) as higher compared to CXT− (Acquisition 1: *M* = 38.86, *SD* = 31.31; Acquisition 2: *M* = 19.87, *SD* = 26.89), *F*_(1, 78)_ = 66.42, *p* < 0.001, η^2^_*p*_ = 0.46, and *F*_(1, 78)_ = 316.37, *p* < 0.001, η^2^_*p*_ = 0.80, respectively. However, this difference increased from Acquisition 1 to Acquisition 2, *F*_(1, 78)_ = 58.48, *p* < 0.001, η^2^_*p*_ = 0.43, indicating successful contextual fear conditioning (see Figure 6). The Context × *NPSR1* × *5HTT*LPR interaction indicated that although all four combined genotype groups reported higher US-expectancy in CXT+ compared to CXT− across both acquisition phases (all *p*s < 0.001), S+/AA carriers displayed a greater difference in expectancy ratings between CXT+ (*M* = 91.00, *SD* = 12.55) and CXT− (*M* = 20.25, *SD* = 18.19) compared to all other combined genotype groups (all *p*s ≤ 0.05).

#### Correlation analysis

**Figure 7:**
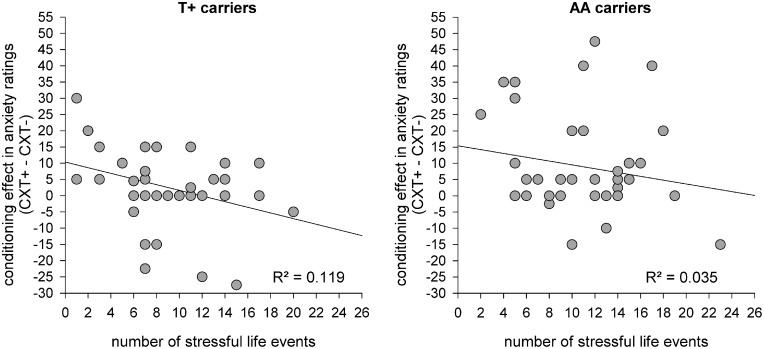
**Scatterplots for correlations between conditioning effects in anxiety ratings on Day 1 (difference score: CXT+ - CXT−) and the number of stressful life events for *NPSR1* genotype groups: risk allele carriers T+ (left) and no risk allele carriers AA (right)**.

To elucidate the interaction between genotype and life stress on contextual fear conditioning and shed light on the absent conditioning effect regarding anxiety ratings in T+ allele carriers, correlations with the number of stressful life events were calculated, as stressful events were found to modulate the effects of *5HTT*LPR and *NPSR1* polymorphisms on levels of anxiety (Klauke et al., 2011, 2012; Klucken et al., 2013). To this end, contextual fear conditioning effects were assessed as the difference between anxiety responses in CXT+ and CXT−. These difference scores for startle and rating data were then correlated with the number of stressful life events reported by the participants. For startle data, four correlation analyses were carried out separately for each combined genotype group (S+/T+, S+/AA, LL/T+, LL/AA) since the interaction between both genotypes influenced startle data. For anxiety ratings correlation analyses were conducted separately for each *NPSR1* genotype group (T+, AA), irrespective of the *5HTT*LPR genotype because it had no influence on rating data. Results showed no significant correlations between conditioning effects in startle data and the number of stressful life events in any genotype group (all *p*s > 0.1). In contrast, the difference between anxiety ratings for CXT+ vs. CXT− correlated negatively with the number of stressful life events in the T+ allele group (*r* = −0.345, *p* = 0.032) but not in the AA allele group (*r* = −0.186, *p* = 0.251). Thus, in the T+ allele group an increase in the number of experienced life events was associated with a decrease in contextual fear conditioning as reflected in anxiety ratings (see Figure 7).

### Extinction (Day 2)

#### Fear-potentiated startle

**Figure 8:**
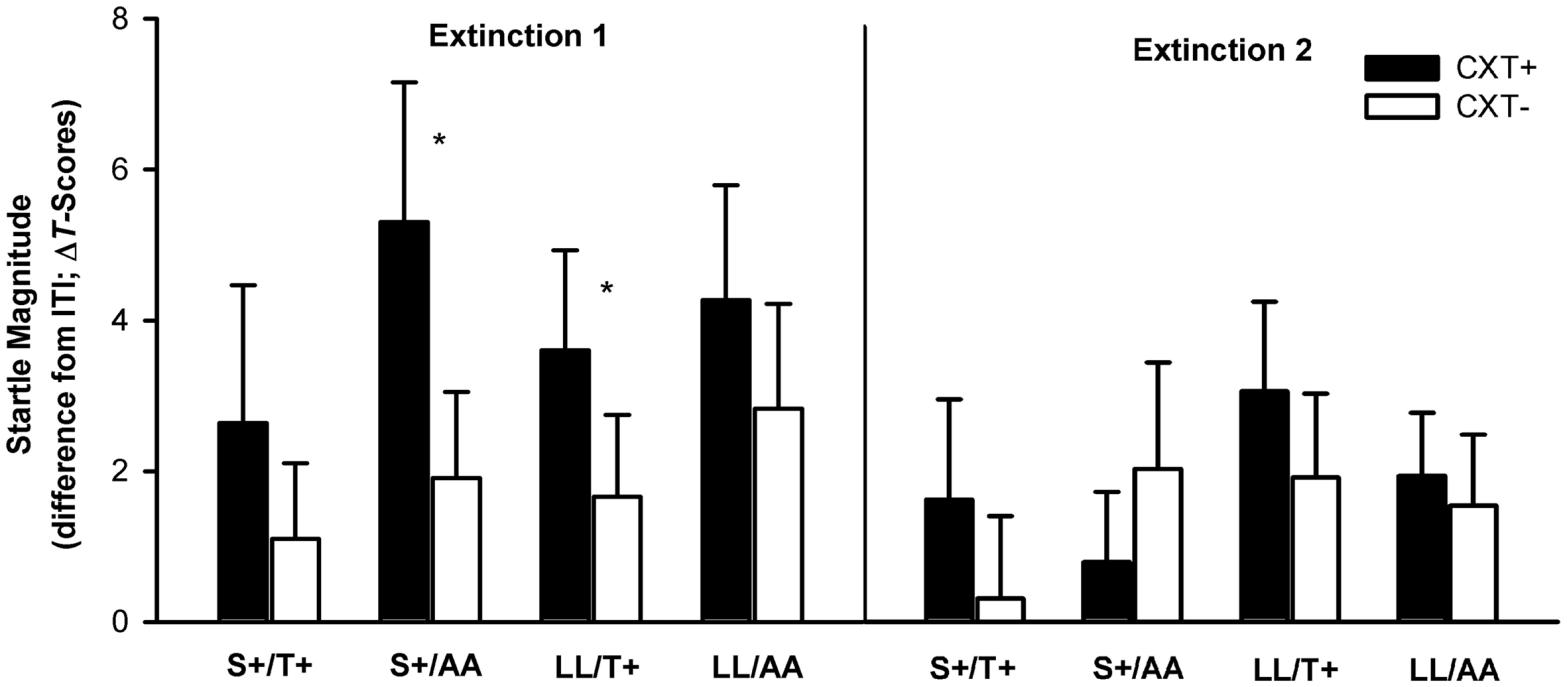
**Startle magnitudes during Extinction1 (left) and Extinction 2 (right) on Day 2.** Black bars depict differences between startle magnitudes during CXT+ (anxiety context) and ITI (intertrial–interval, corridor). White bars depict differences between startle magnitudes during CXT− (safety context) and ITI. Results are shown separately for each combined genotype group of *5HTT*LPR (S+ vs. LL) and *NPSR1* (T+ vs. AA) polymorphisms. Error bars represent standard error of the mean (SEM). ^*^p ≤ 0.05.

The ANOVA revealed a significant main effect of context, *F*_(1, 76)_ = 5.94, *p* = 0.017, η^2^_*p*_ = 0.07, and a significant interaction of Phase × Context, *F*_(1, 76)_ = 6.17, *p* = 0.015, η^2^_*p*_ = 0.08, indicating successful extinction. While startle magnitudes were higher in CXT+ (*M* = 3.96, *SD* = 7.18) compared to CXT− (*M* = 1.93, *SD* = 5.46), *F*_(1, 79)_ = 10.49, *p* = 0.002, η^2^_*p*_ = 0.12, during Extinction 1, this effect lost significance during Extinction 2, CXT+ (*M* = 1.94, *SD* = 5.03) and CXT− (*M* = 1.59, *SD* = 5.28), *F*_(1, 79)_ < 1. There were no significant interaction effects involving any genotype (all *p*s > 0.1). Nevertheless, since we found a modulation of both genotypes on the acquisition of fear-potentiated startle, as an exploratory operation we analyzed the time course of extinction of the four genotype groups separately (see Figure 8). During Extinction 1, carriers of only one risk allele (S+ *or* T+ i.e., groups S+/AA and LL/T+) showed higher startle magnitudes in CXT+ compared to CXT−, *F*_(1, 19)_ = 5.84, *p* = 0.026, η^2^_*p*_ = 0.24, and *F*_(1, 19)_ = 4.37, *p* = 0.050, η^2^_*p*_ = 0.19, respectively, whereas carriers of both risk alleles (S+/T+) and no risk allele (LL/AA) did not (all *p*s > 0.3). All four genotype groups extinguished fear-potentiated startle during Extinction 2 (all *p*s > 0.2).

#### Skin conductance

SCL decreased from Extinction 1 (*M* = 0.635, *SD* = 0.235) to Extinction 2 (*M* = 0.622, *SD* = 0.228), *F*_(1, 76)_ = 5.78, *p* = 0.019, η^2^_*p*_ = 0.07, (main effect of phase). Additionally, there was a marginally significant interaction of Context × Phase, *F*_(1, 76)_ = 3.50, *p* = 0.065, η^2^_*p*_ = 0.04, indicating overall successful extinction. During Extinction 1, SCL was higher in CXT+ (*M* = 0.638, *SD* = 0.236) compared to CXT− (*M* = 0.632, *SD* = 0.234), *F*_(1, 79)_ = 6.15, *p* = 0.015, η^2^_*p*_ = 0.07, but this difference disappeared during Extinction 2, *F*_(1, 79)_ < 1, (CXT+: *M* = 0.621, *SD* = 0.228; CXT−: *M* = 0.623, *SD* = 0.230, see Figure 4). There was also a significant main effect of *5HTT*LPR genotype, *F*_(1, 76)_ = 5.48, *p* = 0.022, η^2^_*p*_ = 0.07, due to LL carriers having higher overall SCL during extinction (*M* = 0.688, *SD* = 0.222) compared to S+ carriers (*M* = 0.569, *SD* = 0.226).

#### Anxiety rating

**Figure 9:**
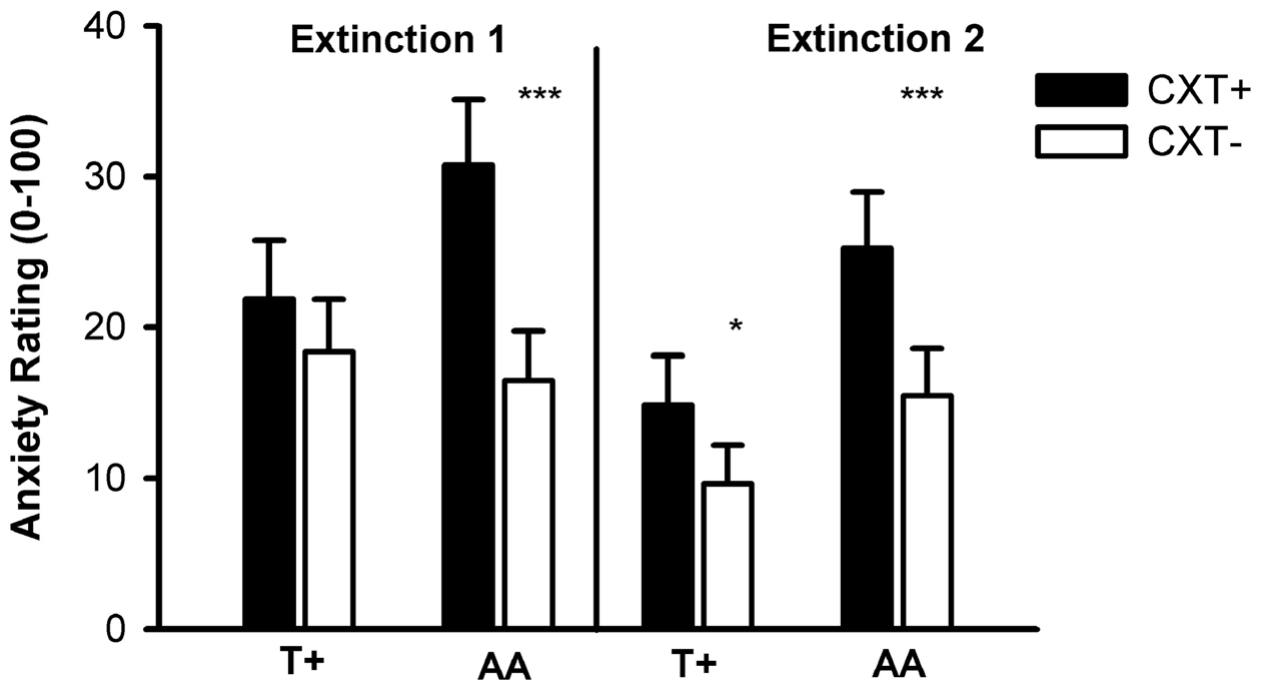
**Anxiety ratings from 0 (no anxiety at all) to 100 (very high anxiety) after Extinction 1 (left) and Extinction 2 (right) on Day 2.** Black bars depict ratings for CXT+ (anxiety context, paired with unconditioned stimulus). White bars depict ratings for CXT− (safety context). Results are shown separately for *NPSR1* genotype groups (T+ vs. AA carriers). Error bars represent standard error of the mean (SEM). ^*^p < 0.05, ^***^p ≤ 0.001.

The ANOVA revealed significant main effects of phase, *F*_(1, 76)_ = 13.60, *p* < 0.001, η^2^_*p*_ = 0.15, and context, *F*_(1, 76)_ = 21.60, *p* < 0.001, η^2^_*p*_ = 0.22, and significant interactions of Context × *NPSR1*, *F*_(1, 76)_ = 4.71, *p* = 0.033, η^2^_*p*_ = 0.06, and Phase × Context × *NPSR1*, *F*_(1, 76)_ = 3.93, *p* = 0.051, η^2^_*p*_ = 0.05. Contrasts regarding the three-way interaction showed that AA carriers reported higher anxiety ratings for CXT+ compared to CXT− after both Extinction 1, *F*_(1, 39)_ = 18.88, *p* < 0.001, η^2^_*p*_ = 0.33, and Extinction 2, *F*_(1, 39)_ = 16.39, *p* < 0.001, η^2^_*p*_ = 0.30. In contrast, T+ carriers only reported higher anxiety for CXT+ compared to CXT− after Extinction 2, *F*_(1, 39)_ = 4.18, *p* = 0.048, η^2^_*p*_ = 0.10, but anxiety ratings for CXT+ after Extinction 2 were higher in AA compared to T+ carriers, *F*_(1, 78)_ = 4.39, *p* = 0.039, η^2^_*p*_ = 0.05, (see Figure 9).

#### US-expectancy rating

There were significant main effects of phase, *F*_(1, 76)_ = 16.14, *p* < 0.001, η^2^_*p*_ = 0.18, and context, *F*_(1, 76)_ = 112.56, *p* < 0.001, η^2^_*p*_ = 0.60, and significant interactions of Phase × Context, *F*_(1, 76)_ = 27.11, *p* < 0.001, η^2^_*p*_ = 0.26, and Context × *NPSR1*, *F*_(1, 76)_ = 4.38, *p* = 0.040, η^2^_*p*_ = 0.06. AA carriers reported higher US-expectancy for CXT+ (*M* = 63.91, *SD* = 19.84) compared to T+ carriers (*M* = 51.48, *SD* = 24.28) after the extinction phases, *F*_(1, 78)_ = 6.29, *p* = 0.014, η^2^_*p*_ = 0.08. Nevertheless AA, *F*_(1, 39)_ = 86.56, *p* < 0.001, as well as T+ carriers, *F*_(1, 39)_ = 35.54, *p* < 0.001, η^2^_*p*_= 0.48, reported higher US-expectancy in CXT+ compared to CXT− (AA: *M* = 25.26, *SD* = 24.85; T+: *M* = 25.56, *SD* = 27.08). *Post-hoc* contrasts regarding the Phase × Context interaction revealed that US-expectancy for CXT+ was rated as higher than for CXT− after both extinction phases (all *p*s < 0.001), but the difference between ratings for CXT+ and CXT− decreased from Extinction 1 to Extinction 2, *F*_(1, 79)_ = 27.48, *p* < 0.001, η^2^_*p*_ = 0.26, thus indicating extinction.

## Discussion

The modulation of contextual fear conditioning and extinction by *5HTT*LPR and *NPSR1* polymorphisms were investigated with a VR paradigm with two offices rooms as conditioned contexts. Human as well as animal research suggests that the T allele of the *NPSR1* polymorphism (Pape et al., 2010; Raczka et al., 2010; Domschke et al., 2011) and the S allele of the *5HTT*LPR polymorphism (Canli and Lesch, 2007; Lonsdorf et al., 2009; Klumpers et al., 2012) are vulnerability factors for enhanced anxiety levels and anxiety disorders, presumably as a result of facilitated fear conditioning (Orr et al., 2000; Mineka and Oehlberg, 2008). Since contextual fear conditioning is an important model of sustained anxiety and as a characteristic of anxiety disorders, we expected that carriers of these two risk alleles would exhibit facilitated contextual fear conditioning.

First and most important, we found that contextual fear conditioning, as measured with the “non-cognitive” behavioral measure of fear-potentiated startle, is modulated by an interaction of the *NPSR1* and the *5HTT*LPR polymorphisms. Only participants carrying both risk alleles (S+/T+) showed enhanced startle responses in the anxiety compared to the safety context during conditioning. Since this effect was especially clear in the later acquisition phase we conclude that it reflects learning by experience. The fear-potentiated startle reflex is preserved across species and used as a translational measure of fear. This response reflects the activation of the innate defensive system mediated by the amygdala which is especially relevant for implicit and automatic fear learning (Mineka and Öhman, 2002; Hamm and Weike, 2005). Therefore, the gene × gene interaction on the fear-potentiated startle reflex further underscores the importance of both polymorphisms and transmitter systems in amygdala-dependent fear learning. Furthermore, this heightened behavioral expression of conditioned contextual anxiety in carriers of the S+ and the T+ allele might function as an endophenotype of anxiety disorders, particularly those characterized by sustained anxiety levels. Supporting this view, firstly studies by Grillon et al. (2008, 2009) revealed that panic disorder and PTSD are characterized by enhanced contextual anxiety, as indicated by fear-potentiated startle. Secondly, disease-specific genetic associations between *5HTT*LPR and PTSD (Kolassa et al., 2010; Wang et al., 2011) and between *NPSR1* and panic disorder (Domschke et al., 2011) were reported. Interestingly, 24 h after consolidation of the fear memory, only carriers of one risk allele (S+/AA, T+/LL) exhibited conditioned startle discrimination, whereas the fear-potentiated startle was already extinguished in carriers of both risk alleles. Carrying both risk alleles not only seems to facilitate fear learning but also to speed up fear extinction on a behavioral level. In contrast, carrying one risk allele seems to delay the expression of contextual fear.

Second, our results indicate successful contextual fear conditioning as reflected in enhanced physiological arousal (SCL, Figure 4) in the anxiety context compared to the safety context. Skin conductance effects are frequently interpreted as a reliable indicator of successful learning in cued (Olsson and Phelps, 2004; Schiller et al., 2010; Tabbert et al., 2011) as well as contextual fear conditioning (Tröger et al., 2012; Glotzbach-Schoon et al., 2013). However, we found no modulation of this conditioning effect by the examined genetic polymorphisms. SCL did also not differ between genotype groups before the experiment (i.e., during the pre-acquisition phase), suggesting an equal arousal level among all participants. Since previous studies on cue conditioning also could not find any modulation of conditioned SCR by *5HTT*LPR or *NPSR1* polymorphisms (Lonsdorf et al., 2009; Raczka et al., 2010; Klucken et al., 2013), it might be concluded that skin conductance is rarely influenced by these genetic variants. Differential skin conductance responses in fear conditioning presumably depend on contingency awareness i.e., the explicitly learned association between CS and US (Hamm and Vaitl, 1996), and participants in the present study were very well aware of the contingencies (see US-expectancy rating, Figure 6). As a matter of fact, US-expectancy ratings and SCL revealed contextual fear conditioning effects already in the first acquisition phase indicating that participants cognitively apprehended contingencies quite early. Therefore, it seems reasonable to conclude that genetic influences on SCL cannot be expected, at least if the contingencies are clear and easily apprehended, as in the present study.

Surprisingly, we found an influence of *NPSR1* but no interaction between both polymorphisms on explicit anxiety ratings. AA carriers (no risk allele) reported higher anxiety in the anxiety context compared to the safety context after contextual fear conditioning. This differential learning effect could still be found during extinction on Day 2. Presumably, as a result of this enhanced conditioning effect in AA carriers, extinction of explicit anxiety ratings was delayed in AA carriers too. Notably, US-expectancy ratings for CXT+ were also higher in AA compared to T+ carriers after extinction. This might be a hint for fast contextual fear conditioning in combination with extinction deficits in AA carriers on a verbal, explicit level. This result stands in contrast to the enhanced conditioning effects of fear-potentiated startle in S+ and T+ carriers in our study.

To explain this finding three points have to be considered. Firstly, a fear response can vary on two levels: an implicit behavioral level (i.e., fear-potentiated startle reflex) vs. an explicit/cognitive level (i.e., verbal ratings) (Hamm and Weike, 2005). These two levels can be influenced independently from another and even dissociate. Diverging responses on explicit and implicit levels have already been reported in the fields of fear extinction (Vansteenwegen et al., 1998) and pain relief learning (Andreatta et al., 2010). Here, we found a dissociation of implicit and explicit levels of fear according to the *NPSR1* genotype. T+ allele carriers (in addition with a S+ allele of the *5HTT*LPR genotype) exhibited fear-potentiated startle but no explicit anxiety, whereas AA allele carriers showed no fear-potentiated startle but reported explicit anxiety. In any case, our results emphasize the importance of measuring different fear levels.

Secondly, it should be noted that AA compared to T+ carriers reported higher arousal triggered by the US. This difference in the explicit evaluation of the US might have contributed to the differential conditioning effects in explicit anxiety ratings in AA but not T+ carriers. To confirm whether US-arousal was associated with anxiety ratings and not startle data, we correlated differential conditioning effects in anxiety ratings and startle data with US-arousal. Interestingly, we found a significant positive correlation between US-arousal and the amount of differential conditioning in anxiety ratings but not with differential conditioning effects in fear-potentiated startle^1^. Thus, US-arousal might have had a greater impact on the explicit level than on the implicit fear-potentiated startle response.

Thirdly, an interaction between stress and the NPS system has been reported in two rodent (Ebner et al., 2011; Jüngling et al., 2012) and one human study (Klauke et al., 2012). In line with this research, in our study the conditioning effect in explicit anxiety ratings was not only influenced by *NPSR1* genotype but additionally by the amount of stressful life events. In detail, there was a negative correlation between the contextual fear conditioning effect on the explicit anxiety level and the number of stressful life events. This negative association could only be found in T+ carriers, meaning that the higher the number of stressful life events is the weaker is the conditioning effect. T+ risk allele carriers with many life events even tended to rate the safety context as more anxiety inducing than the anxiety context. Notably, not only faster and higher fear conditionability is discussed as a diathesis for anxiety disorders (Orr et al., 2000), but also the failure to inhibit fear responses in the presence of safety (Lissek et al., 2005, 2009). Carrying the T risk allele in addition to high amounts of life stress might impair safety learning on a cognitive explicit level. However, this is very speculative as participants were not pre-selected on the basis of life events and this negative association has to be replicated in larger samples.

A limitation of our study might be that fear-potentiated startle effects were not very strong and could not be seen across all 80 participants but only in the high risk subgroup. The reasons for this discrepancy might be that we did not realize enough learning trials or that the US was not aversive enough. However, our paradigm was effective enough to evoke fast contextual anxiety in carriers of the two risk alleles for anxiety disorders, whereas carriers of only one risk allele showed delayed fear expression. We suggest that future studies should use our paradigm to examine a more anxious sample perhaps revealing stronger conditioning effects (Glotzbach-Schoon et al., 2013) especially on the cognitive level in T+ allele carriers as well.

In summary, we found an effect of both risk alleles of the *5HTT*LPR and the *NPSR1* polymorphisms on the acquisition of contextual fear measured with an implicit behavioral measure, the fear-potentiated startle. On an explicit level, the examined *5HTT*LPR polymorphism had no effect on anxiety ratings. Only the no risk allele carriers of the *NPSR1* genotype exhibited differential contextual fear conditioning and extinction deficits on an explicit level. The serotonin system might only modulate amygdala-dependent fear learning but not the explicit evaluation of a threatening context, whereas the NPS system might have opposing effects on explicit and implicit anxiety responses. Further studies are needed to elucidate the role of the *NPSR1* in explicit and implicit contextual fear conditioning. However, we demonstrated that both genetic polymorphisms play an important role in contextual fear conditioning which is a model for unpredictable threat and sustained anxiety characteristic for panic disorder or PTSD. In conclusion, enhanced contextual fear conditioning may function as an endophenotype for these anxiety disorders.

### Conflict of interest statement

Prof. Paul Pauli is shareholder of a commercial company that develops virtual environment research systems for empirical studies in the field of psychology, psychiatry, and psychotherapy. Prof. Andreas Mühlberger is shareholder and executive officer of the same company. The other authors declare that the research was conducted in the absence of any commercial or financial relationships that could be construed as a potential conflict of interest.

## Footnotes

^1^Differential conditioning effects in anxiety ratings and startle data were assessed as the difference score between anxiety ratings/startle response in CXT+ and CXT− at Day 1. Correlation between anxiety ratings and US-arousal: *r* = 0.223, *p* = 0.047; and between startle response and US-arousal: *r* = 0.074, *p* = 0.512.
